# MiR-21 as prognostic biomarker in head and neck squamous cell carcinoma patients undergoing an organ preservation protocol

**DOI:** 10.18632/oncotarget.14253

**Published:** 2016-12-27

**Authors:** Lidia Maria Rebolho Batista Arantes, Ana Carolina Laus, Matias Eliseo Melendez, Ana Carolina de Carvalho, Bruna Pereira Sorroche, Pedro Rafael Martins De Marchi, Adriane Feijó Evangelista, Cristovam Scapulatempo-Neto, Luciano de Souza Viana, André Lopes Carvalho

**Affiliations:** ^1^ Molecular Oncology Research Center, Barretos Cancer Hospital, Barretos, SP, Brazil; ^2^ Department of Clinical Oncology, Barretos Cancer Hospital, Barretos, SP, Brazil; ^3^ Department of Pathology, Barretos Cancer Hospital, Barretos, SP, Brazil; ^4^ Department of Head and Neck Surgery, Barretos Cancer Hospital, Barretos, SP, Brazil

**Keywords:** HNSCC, chemoradiation, microRNA

## Abstract

Despite progress in the treatment of head and neck squamous cell carcinoma (HNSCC) in recent decades, including new surgical techniques, radiotherapy advances and chemotherapy schedules, the prognosis for the affected patients has not improved at the same pace, and still, most HNSCC patients are diagnosed in advanced stages. To increase their survival, the development of better screening methods for early detection is required and appropriate tailored therapeutic interventions are desired. The aim of the present study was to evaluate miRNAs as prognostic biomarkers in patients undergoing organ preservation protocol for locally advanced HNSCC. For this purpose, we assessed the global miRNA expression profile of 15 HNSCC patients (‘screening set’) to identify miRNAs differentially expressed in responders and non-responders to therapy. Four miRNAs differentially expressed in HNSCC samples from the ‘screening set’ were validated in a different cohort of patients (47 samples - ‘validation set’). The results from the ‘validation set’ showed that the higher expression of one of these miRNAs, miR-21, was negatively associated with the treatment response to the organ preservation protocol (p=0.029). A multivariate analysis showed that, in a model adjusted for age, tumor site, p16 immunoexpression and tumor resectability, high expression of miR-21 remained an independent predictor of poor response to the organ preservation protocol (OR=5.69; 95%CI 1.27-25.58; p=0.023), together with clinical stage IV (OR=5.05; 95%CI 1.22-20.88; p=0.025). Furthermore, considering the entire cohort, patients with high expression of miR-21 had worse survival. A multivariate Cox regression analysis also showed miR-21 (HR=2.05; 95%CI 1.05-4.02; p=0.036) and clinical stage IV (HR=3.17; 95%CI 1.49-6.77; p=0.003) as independent prognostic factors (model adjusted for age, tumor site, tumor resectability, and sets ‘screening’ or ‘validation’).

In conclusion, the results of this study suggest that the evaluation of miR-21 expression could be an important tool for treatment planning and a prognosis predictior for HNSCC patients undergoing organ preservation protocols.

## INTRODUCTION

The worldwide annual incidence of head and neck squamous cell carcinoma (HNSCC) is approximately 740,000 cases, with approximately 300,000 deaths each year [1, 2]. This high incidence, mainly affecting the oral cavity, the pharynx and the larynx, is associated with low survival and high mortality rates.

Over the last 30 years, much has been learned regarding the role of chemotherapy, radiation therapy, and combined-modality treatment with chemoradiation (CRT) in the treatment of locally advanced head and neck cancer. For instance, taxanes have been proven to have significant single-agent activity, increasing interest in the incorporation of taxanes into induction regimens for the treatment of patients with locally advanced HNSCC [3].

The treatment of early stage HNSCC tumors is conducted preferably with surgery and radiation, with curative rates of approximately 80 to 90%. Conversely, the treatment of patients with locally advanced disease (stage III or IV) involves CRT or surgery followed by adjuvant radiotherapy/CRT. However, only 30-50% of patients with locally advanced disease survive more than five years, despite the advances in surgical techniques and the recognized benefits of CRT [4–6].

Furthermore, despite recent improvements in treatment approaches, the prognosis for HNSCC patients did not improve at the same pace as these advancements, and still, most of the cases are diagnosed in advanced stages of HNSCC. In this scenario, improvement in patient's survival requires both early detection [7] as well as the possibility of predicting, at the moment of diagnosis, which patients will benefit from a specific treatment. Tailoring the treatment is of great importance and may help in the management of treatment planning and follow-up, improving survival rates.

MicroRNAs (miRNAs) represent a class of non-coding RNAs acting as post-transcriptional gene expression regulators by inhibiting translation or destabilizing mRNAs. They are involved in the regulation and coordination of multiple cellular pathways and processes. MiRNAs are responsive to various cellular stressors and are key players in many diseases including cancer [8, 9]. Studies have shown that miRNAs may affect cancer development and the regulation of the immune/inflammation or cell death responses as well as may be used in early detection, diagnosis and prognosis of different diseases [10].

The aim of the present study was to evaluate if miRNAs are able to predict the response to an organ preservation protocol based on chemoradiation [3]. For this purpose, we assessed the global miRNA expression profile of HNSCC patients with locally advanced and unresectable tumors undergoing treatment. We looked for miRNAs differentially expressed in complete responders vs. non-responders, thus serving as markers to predict response.

## RESULTS

### Characteristics of the patients

The study population comprised a cohort of 71 patients with a confirmed diagnosis of squamous cell carcinoma of the oropharynx, larynx or hypopharynx and submitted to an organ preservation protocol in our institution from 2009 to 2011. The protocol is based on induction chemotherapy followed by concomitant chemoradiation; details of the treatment protocol have been previously published [3]. Patients were mainly males (95.8%), with an age range of 40-76 years (median of 56 years). Tobacco or alcohol consumption was reported by 80.3% and 38.0% of the patients, respectively. The primary tumor sites were located in the oropharynx (49.3%), larynx (39.4%) and hypopharynx (11.3%). Clinical stage (CS) classification was T2/T3 in 46 cases (64.8%) and T4 in 25 cases (35.2%), N0/N1 in 35 cases (49.3%) and N2/N3 in 36 cases (50.7%). Patients were grouped as 29 cases with CS III (40.8%) and 42 with CS IV (59.2%). Six cases were considered unresectable (8.5%). With regard to HPV status, 6 (18.2%) of the oropharynx tumors were considered HPV-positive. Only 1 patient with a larynx tumor (3.6%) and none of the patients with hypopharynx tumors presented with positive p16 immunoexpression.

### miRNA microarrays expression (‘screening set’)

The miRNA expression profile for patients who underwent chemoradiation protocol was assessed in 15 samples (‘screening set’) by microarray. FFPE samples were divided into the following 2 groups: 8 FFPE samples from responder patients and 7 FFPE samples from non-responder patients, after chemoradiation. To evaluate differentially expressed miRNAs between the two groups, the rank product analysis was performed for p-value ≤ 0.001, in tandem with the rate of false positive predictions (pfp) ≤ 0.05.

**Figure 1:**
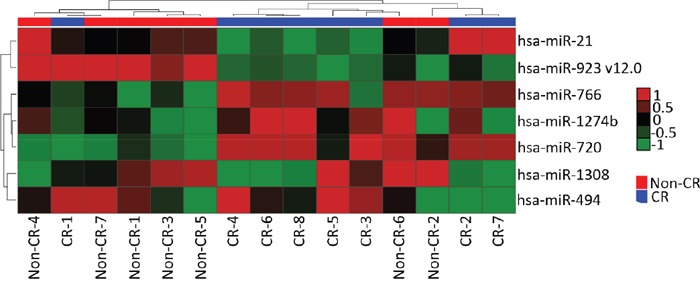
The hierarchical clustering image of 7 differentially expressed miRNAs (p≤0.001) in HNSCC when comparing groups of Complete Response (CR) versus Partial Response + Stable Disease + Tumor Progression (Non-CR)

The heatmap of the differentially expressed miRNAs with p-value ≤ 0.001 identified 7 miRNAs (hsa-miR-21, hsa-miR-923_v12.0, hsa-miR-766, hsa-miR-1274b, hsa-miR-720, hsa-miR-1308 and hsa-miR-494; Figure 1). Of the 7 significantly different miRNAs, 4 miRNAs (hsa-miR-21, hsa-miR-923_v12.0, hsa-miR-720 and hsa-miR-494) were selected for further validation in the ‘validation set’.

### miRNAs RT-qPCR expression (‘validation set’)

The validation of microarray results was conducted by reverse transcription quantitative PCR (RT-qPCR). The data were normalized using the expression of SNORD48 (RNU48) and U6snRNA as endogenous references.

The miRNA expression was then validated in a different set of patients (‘validation set’). The ‘validation set’ included 47 patients from the remaining cohort, who had finished the organ preservation protocol and were evaluated for treatment response. The expression levels of the four selected miRNAs (miR-21, miR-923, miR-720 and miR-494) were assessed.

When evaluating complete responders after chemoradiation (CRT) vs. non-responders (partial response or stable disease or tumor progression), only the results for miR-21 were validated: of all the patients who highly expressed miR-21, 70.8% did not have a complete response after chemoradiation, while 39.1% had a complete response (p=0.029) (Table 1). Moreover, in a multivariate analysis, using a model adjusted for age, tumor site, p16 immunoexpression and tumor resectability, high expression of miR-21 remained as an independent predictor of poor response to the organ preservation protocol (OR=5.69; 95%CI 1.27-25.58; p=0.023), together with clinical stage IV (OR=5.05; 95%CI 1.22-20.88; p=0.025) (Table 2).

**Table 1:** Results from univariate analysis for organ preservation protocol response and microRNA expression (qPCR) for the ‘validation set’

	*Overall response after chemoradiation (CRT)*		
	Responders (CR)n (%)	Non-responders (PR+SD+TP)n (%)	p-value
**miR-21**			
*Low expression*	14 (60.9)	7 (29.2)	**0.029**
*High expression*	9 (39.1)	17 (70.8)	
**miR-494**			
*Low expression*	10 (43.5)	9 (37.5)	0.676
*High expression*	13 (56.5)	15 (62.5)	
**miR-720**			
*Low expression*	12 (52.2)	10 (41.7)	0.471
*High expression*	11 (47.8)	14 (58.3)	
**miR-923**			
*Low expression*	8 (34.8)	9 (37.5)	0.846
*High expression*	15 (65.2)	15 (62.5)	

CR – complete response; PR – partial response; SD – stable disease; TP – tumor progression.

**Table 2:** Logistic regression analysis for poor overall response to the organ preservation protocol (for the ‘validation set’ patients)

Variable	Adjusted OR* (95% CI)	p-value
***miR-21expression***		
*Low expression*	1 (ref.)	**0.023**
*High expression*	5.69 (1.27 – 25.58)	
***Tumor Staging***		
*CS III*	1 (ref.)	**0.025**
*CS IV*	5.05 (1.22 – 20.88)	

*Adjusted by age, tumor site, p16 immunoexpression and tumor resectability

### Expression profile and clinical data associations

The expression profile of the 4 selected miRNAs was analyzed considering the entire cohort (all patients with clinical information and samples analyzed for miRNAs by RTq-PCR) for potential associations with patients’ clinical-pathological characteristics and survival analysis.

The analysis showed that the patients who were alcohol consumers had higher miR-21 expression than the patients who did not drink alcohol (p=0.009). Moreover, oropharynx patients had higher miR-720 expression when compared to larynx and hypopharynx patients (p=0.043) (Table 3).

**Table 3:** Results of univariate analysis of clinical, pathological and microRNA expression data of the patients (entire cohort) enrolled in the study

	miR-21 Expression			miR-494 Expression			miR-720 Expression			miR-923 Expression		
	Low n (%)	High n (%)	p-value	Low n (%)	High n (%)	p-value	Low n (%)	High n (%)	p-value	Low n (%)	High n (%)	p-value
***Age***												
< 60 years-old	21 (47.7)	23 (52.3)	0.736	19 (43.2)	25 (56.8)	0.311	19 (43.2)	25 (56.8)	0.188	23 (52.3)	21 (47.7)	0.522
≥ 60 years-old	14 (51.9)	13 (48.1)		15 (55.6)	12 (44.4)		16 (59.3)	11 (40.7)		12 (44.4)	15 (55.6)	
***Gender***												
Male	33 (48.5)	35 (51.5)	0.614*	32 (47.1)	36 (52.9)	0.604*	34 (50.0)	34 (50.0)	1.000*	33 (48.5)	35 (51.5)	0.614*
Female	2 (66.7)	1 (33.3)		2 (66.7)	1 (33.3)		1 (33.3)	2 (66.7)		2 (66.7)	1 (33.3)	
***Tobacco consumption***												
Yes	26 (45.6)	31 (54.4)	0.211	27 (47.4)	30 (52.6)	0.860	30 (52.6)	27 (47.4)	0.257	27 (47.4)	30 (52.6)	0.512
No	9 (64.3)	5 (35.7)		7 (50.0)	7 (50.0)		5 (35.7)	9 (64.3)		8 (57.1)	6 (42.9)	
***Alcohol consumption***												
Yes	8 (29.6)	19 (70.4)	**0.009**	11 (40.7)	16 (53.3)	0.345	13 (48.1)	14 (51.9)	0.880	11 (40.7)	16 (53.3)	0.259
No	27 (61.4)	17 (38.6)		23 (52.3)	21 (47.7)		22 (50.0)	22 (50.0)		24 (54.5)	20 (45.5)	
***Tumor site***												
Oropharynx	17 (48.6)	18 (51.4)	0.904	13 (37.1)	22 (62.9)	0.074	13 (37.1)	22 (62.9)	**0.043**	15 (42.9)	20 (57.1)	0.285
Larynx/Hypopharynx	18 (50.0)	18 (50.0)		21 (58.3)	15 (41.7)		22 (61.1)	14 (38.9)		20 (55.6)	16 (44.4)	
***Ressecability***												
Resectable	32 (49.2)	33 (50.8)	1.000*	31 (47.7)	34 (52.3)	1.000*	34 (52.3)	31 (47.7)	0.199*	31 (47.7)	34 (52.3)	0.429*
Unresectable	3 (50.0)	3 (50.0)		3 (50.0)	3 (50.0)		1 (16.7)	5 (83.3)		4 (66.7)	2 (33.3)	
***T Stage***												
T2 and T3	24 (54.2)	22 (47.8)	0.511	21 (45.7)	25 (54.3)	0.609	26 (56.5)	20 (43.5)	0.099	23 (50.0)	23 (50.0)	0.872
T4	11 (44.0)	14 (56.0)		13 (52.0)	12 (48.0)		9 (36.0)	16 (64.0)		12 (48.0)	13 (52.0)	
***N Stage***												
N0 and N1	16 (45.7)	19 (54.3)	0.552	15 (42.9)	20 (57.1)	0.403	20 (57.1)	15 (42.9)	0.192	15 (42.9)	20 (57.1)	0.285
N2 and N3	19 (52.8)	17 (47.2)		19 (52.8)	17 (47.2)		15 (41.7)	21 (58.3)		20 (55.6)	16 (44.4)	
***Tumor Staging***												
CS III	14 (48.3)	15 (51.7)	0.886	10 (34.5)	19 (65.5)	0.060	18 (62.1)	11 (37.9)	0.074	12 (41.4)	17 (58.6)	0.268
CS IV	21 (50.0)	21 (50.0)		24 (57.1)	18 (42.9)		17 (40.5)	25 (59.5)		23 (54.8)	19 (45.2)	
***p16 Immunoexpression***												
Positive	2 (28.6)	5 (71.4)	0.431*	3 (42.9)	4 (57.1)	1.000*	3 (42.9)	4 (57.1)	0.710*	4 (57.1)	3 (42.9)	0.710*
Negative	31 (50.0)	31 (50.0)		30 (48.4)	32 (51.6)		32 (51.6)	30 (48.4)		30 (48.4)	32 (51.6)	

CS – clinical stage

*Fisher’s exact test

**Figure 2:**
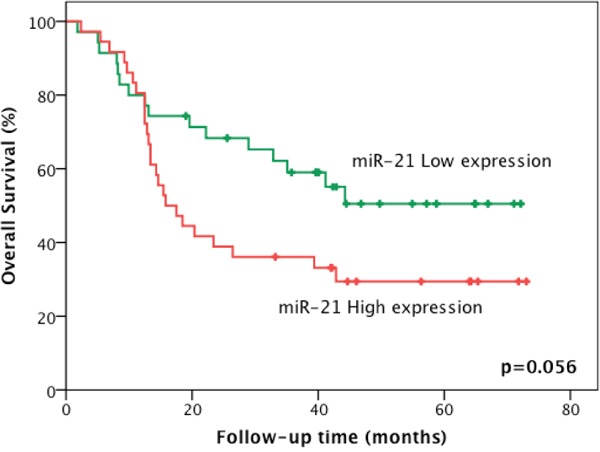
A 5-year overall survival rate according to miR-21 expression level in HNSCC samples

The 5-year disease-free survival (DFS) rate for the cohort was 54.5%, and the 5-year overall survival rate was 40.0%. None of the miRNAs validated in the study were significantly associated with disease-free survival or overall survival in the univariate Kaplan-Meier analysis (Table 4). However, the 5-year overall survival rate for patients with a low expression of miR-21 was 50.5%, compared to a 29.4% overall survival rate for those with a high expression of this miRNA (p=0.056) (Figure 2). Furthermore, a multivariate Cox regression analysis, in a model adjusted for age, tumor site, tumor resectability, and ‘screening’ vs. ‘validation’ sets, indicated that miR-21 (HR=2.05; 95%CI 1.05-4.02; p=0.036) and clinical stage IV (HR=3.17; 95%CI 1.49-6.77; p=0.003) are independent prognostic factors (Table 5).

**Table 4:** Results of univariate Kaplan-Meier survival analysis regarding disease-free and overall survival for the entire cohort

	5-year Disease-Free Survival (%)	p-value(log-rank)	5-year Overall Survival (%)	p-value (log-rank)
***miR-21***				
Low expression	62.7	0.451	50.5	0.056
High expression	43.7		29.4	
***miR-494***				
Low expression	59.5	0.527	43.3	0.683
High expression	50.3		37.9	
***miR-720***				
Low expression	64.4	0.282	45.7	0.376
High expression	45.5		35.0	
***miR-923***				
Low expression	50.8	0.567	39.3	0.489
High expression	58.0		41.3	

**Table 5:** Multivariate Cox regression analysis for overall survival (entire cohort of patients)

Characteristic	Adjusted HR* (95% CI)	p-value
***miR-21expression***		
*Low expression*	1 (ref.)	**0.036**
*High expression*	2.05 (1.05 – 4.02)	
***Tumor Staging***		
*CS III*	1 (ref.)	**0.003**
*CS IV*	3.17 (1.49 – 6.77)	

* Adjusted by age, tumor site, tumor resectability, and set ‘screening vs. validation’

## DISCUSSION

There is still uncertainty concerning the functional role of most miRNAs, as one miRNA may target multiple mRNAs and one mRNA can be regulated by a number of different miRNAs [7, 9, 11–13].

Evidence suggests that miRNAs may both prove to be useful as diagnostic and prognostic markers as well as highlight the competency for targeted therapies [7, 9, 11, 12, 14]. Microarray and RT-qPCR have been widely applied for the identification of miRNAs that are differentially expressed in HNSCC. However, it has been challenging to characterize a consistent miRNA signature. Therefore, assessing global miRNA expression and its association with tumor characteristics could represent a promising approach to improve disease diagnosis and therapeutic protocols and, thus, increase patient survival.

MiR-21 is regarded as a key oncogenic factor and its function in the progression of cancer has been widely investigated in many types of cancers [15, 16]. In many tumors, highly expressed miR-21 is involved in the differentiation, proliferation and apoptosis of the cells, mostly through regulation of the expression of genes related to these processes, therefore playing a part in promoting the genesis and development of tumors [17, 18]. Our data showed that 70.4% of the alcohol-using patients had a higher miR-21 expression when compared to the patients who did not drink alcohol (this analysis included all HNSCC tumors). In contrast to this finding, Hu et al. did not find any correlation between alcohol use and the expression level of miR-21 in larynx tumors [19]. MiR-21 was highly expressed in the larynx [16], pharynx [20], tongue [21], tonsils [22] and in HNSCC [12] when compared to healthy tissue as well as in blood from oral squamous cell carcinoma (OSCC) patients when compared to normal healthy blood controls [17]. Nevertheless, these publications did not relate alcohol consumption to miR-21 expression, possibly because of the lack of information or statistical significance. Regarding to treatment response, 66.7% of the patients who did not have a complete response after chemoradiation had a higher expression of miR-21 when compared to the patients who responded. Corroborating our study, Li et al. also found that tongue tumors with advanced clinical stages, poor differentiation or lymph node metastasis expressed higher levels of miR-21, suggesting that miR-21 is related to cancer progression [23].

MiR-494 is located in human chromosome 14q32 and functions as tumor suppressive or oncogenic miRNA, depending on the tumor type [24, 25]. Low expression of miR-494 has been reported in prostate cancer [26], lung cancer [25] and gastrointestinal tumors [24, 27]. In contrast, miR-494 is up-regulated in colorectal cancer [24, 28]. Interestingly, the genomic region where miR-494 is located has been reported as deleted in 40% of HNSCC patients; these tumors exhibited loss of heterozygosity (LOH) of 14q in at least one allele, and this loss was also correlated with a three-fold increased risk of death [29]. Chang et al. found that miR-494 was down-regulated in HNSCC by microarray, but these data could not be validated by RT-qPCR [20]. Libório-Kimura et al. observed a similar down-regulation in a separate study, where oral cancer tumors were compared to normal healthy tissues [30]. In contrast, OSCC patients’ blood showed a 2-fold up-regulation of miR-494 when compared to healthy control blood [31]. In regard to our study, we did not found any correlation between miR-494 and clinical-pathological characteristics.

MiR-720, originally annotated as a microRNA, has been reclassified as a transfer RNA fragment (tRF). This recently discovered class of small RNAs has been found to be present in diverse organisms at read counts comparable to miRNAs and there is a debate about their biogenesis and function [32–34]. Regardless of its origin and function, miR-720 has been described as a circulating serum biomarker in some tumors, such as colorectal cancers and myelomas [35–37]. MiR-720 expression was significantly up-regulated in cervix squamous cell carcinoma tissue when compared to normal cervix tissue, indicating that miR-720 is closely related to the pathological processes of cervical cancer [38]. Li et al. found that the expression of miR-720 was significantly down-regulated in primary breast cancers, with greater down-regulation in metastatic tumors [39]. Analysis in breast cancers demonstrated that decreased expression of miR-720 was correlated with lymph node metastasis, and re-expression of this miR/tRF in breast cancer cells inhibited cell invasion and migration [39]. Our results showed that 62.9% of the oropharynx patients had a higher miR-720 expression when compared to larynx and hypopharynx patients. To the best of our knowledge, this is the first study that shows a correlation between miR-720 and head and neck cancer.

MiR-923 appears to be a fragment of 28S rRNA. Nevertheless, like miR-720, it has been correlated with survival time. An article comparing bladder cancer patients who received a first-line platinum chemotherapy for locally advanced and/or metastatic carcinoma found that patients with long survival (> 1 year) had a 1.5-fold increased expression of miR-923 compared with patients with short survival (< 1 year) [40]. Zhou et al. showed that miR-923 was up-regulated in Taxol-resistant breast cancer cells, when compared to Taxol-sensitive parental cells [41]. MiR-923 was also down-regulated in lymphocytic leukemia cells [42] and receptive endometrium [43]. We could not confirm findings regarding the correlation of miR-923 with prognosis in our study.

MicroRNAs are promising candidates for use as diagnostic markers because they play an important role in many processes involved in tumor metastasis, have tumor and tissue-specific expression, can be evaluated using a sensitive and quick technique and are suitable to be assessed in FFPE tissues and body fluids. This study presents the evaluation of miRNAs as response predictors and points to a correlation between expression of miR-21 and treatment outcome. Moreover, the high expression of miR-21 in this group of samples is associated with a better 5-year overall survival. The results of this study suggest that the evaluation of miR-21 expression could be an important tool for the management of HNSCC patients, assisting in the stratification of patients who may benefit from an organ preservation protocol and contributing to an improvement in quality of life and survival rates.

## PATIENTS AND METHODS

### Population cohort

The present study included a cohort of 71 patients undergoing organ preservation protocol between 2009 and 2011 at the Department of Head and Neck Surgery, Barretos Cancer Hospital, Barretos, SP, Brazil. Patients were eligible if they had a locally advanced tumor (clinical stage III or IVa-b; M0) of the oropharynx, larynx or hypopharynx that was histologically confirmed as squamous cell carcinoma. Patients should not have been previously submitted to any oncological treatment and have a FFPE sample available for molecular analysis. All patients were required to have measurable disease by Response Evaluation Criteria in Solid Tumors (RECIST, version 1.1), an ECOG-PS ≤ 2 (Eastern Cooperative Oncology Group Performance Status), an age of at least 18 years and adequate liver, renal and bone marrow function. Exclusion criteria included a history of another malignancy, presence of a serious concomitant illness and a psychiatric illness that would preclude the delivery of the treatment. The study was approved by the Barretos Cancer Hospital Institutional Review Board.

### Organ preservation protocol

The organ preservation protocol was based on a protocol used in a phase II clinical trial carried out in our institution; the details of that study were previously published [3]. In summary, the phase II clinical trial was designed to evaluate the feasibility of delivering cisplatin concurrent with radiotherapy after 3 cycles of an induction chemotherapy regimen based on the combination of cisplatin and paclitaxel in advanced HNSCC patients. Patients were monitored every 3 months during the first 2 years and every 6 months thereafter. Physical examination, radiographic disease assessment (CT/MRI scan of head, neck and chest) and fiberoptic endoscopy of the upper digestive tract were performed during regular monitoring or when disease progression/recurrence was suspected.

### FFPE sample processing and RNA purification

H&E sections corresponding to paraffin blocks containing the samples of interest were reviewed by a pathologist to confirm the diagnosis and to characterize the cellular components present. Selected areas in the FFPE samples containing at least 70% of malignant cells were submitted to a total RNA isolation protocol using the Recover All Total Nucleic Acid Isolation kit (*Life Technologies*). The samples were initially treated with xylene, followed by washing twice with absolute ethanol and proteinase K treatment at 50°C for 3 hours. Quantification was performed using a Qubit fluorometer (*Invitrogen*, Carlsbad, CA, USA). RNA was stored at −80°C until use. Due to the scarcity of RNA obtained from FFPE samples, RNA purity or integrity was not evaluated. The amplification of the internal controls (U6 and RNU48) by RT-qPCR was used as indicative of the high quality of the RNA samples.

### miRNA microarrays

The Agilent Human miRNA Microarray (8×15K - G4471A, *Agilent Technologies*) was used in 15 samples from FFPE sections. A total of 100 ng of total RNA was hybridized using miRNA complete labeling and the Hyb Kit (*Agilent Technologies*), according to the manufacturer's instructions. The reactions followed a 2-step preparation, represented by dephosphorylation and denaturation of the total RNA followed by incorporation of the Cy3 fluorochrome in a ligation reaction by the T4 ligase. The next steps included standard washing procedures and hybridization with microarray slides. The images were scanned using an Agilent DNA microarray scanner with SureScan technology (*Agilent Technologies*). Raw data were submitted to ArrayExpress (E-MTAB-4854).

The raw data were obtained using Feature Extraction software v.11.0 (Agilent Technologies) and submitted to R environment v. 3.2.3 [44] for further analysis. The median signals (*gMedianSignal* and *gBGMedianSignal*) were used. Following background subtraction and log2 scale transformation, the normalization was performed using the quantile method with the aroma light package [45]. Differentially expressed microRNAs were obtained by rank product analysis using the RankProd package [46], considering p-values and positive false predictions (pfp) ≤ 0.05. Differentially expressed microRNAs were clustered using the Pearson correlation in a ComplexHeatmaps package [47].

### RT-qPCR validation of selected differentially expressed miRNAs

RT-qPCR validation of selected differentially expressed miRNAs was performed in FFPE samples from a different set of 47 patients (not included in the ‘screening set’). Each assay was conducted using the Taqman MicroRNA Reverse Transcription kit (*Applied Biosystems*) according to the manufacturer's protocol. Briefly, 10 ng of total RNA was reverse-transcribed to cDNA using a MultiScribe Reverse Transcriptase and a stem-loop primer (*Applied Biosystems*) specific for each microRNA, according to manufacturer's instructions. RT-qPCR was performed using a TaqMan PCR kit on 96-well plates in the 7900HT Fast Real-Time PCR System (*Applied Biosystems*). All reactions were performed in triplicate. To evaluate the differential expression of each microRNA, the 2-^ΔCt^ method was employed [47]. Mean Ct values of U6 (assay: U6 snRNA 001973) and RNU48 (assay: RNU48 snRNA 001006) were used for normalization. The miRNA levels measured during the validation step were converted into discrete variables by splitting the samples into two classes (high and low expression) using the ΔCt median level and considering all samples were evaluated as cutoff. The expression assay IDs of the miRNA that were analyzed were as follows: hsa-mir-21 snRNA 000397, hsa-mir-923 snRNA 002153, hsa-mir-720 snRNA 002895 and hsa-mir-494 snRNA 002365.

### p16 immunoexpression

p16 immunoexpression was assessed by immunohistochemistry (IHC) performed on 4 mm thick formalin-fixed paraffin embedded (FFPE) tissue sections, using the CINtec® p16INK4A assay, according to the manufacturer's instructions (CINtec® Histology Kit, Ventana Medical Systems, Inc., Tucson, Arizona, USA). For oropharynx cases only, when p16 staining was observed in ≥ 75% of tumor cells, we would consider the tumor as HPV positive.

### Statistical analysis

The Chi square test and Fisher's exact test were used to evaluate the associations between miRNA expression, tumor response and clinical variables, as appropriate. A multivariate logistic regression was performed to identify the independent variables associated with tumor response in the organ preservation protocol. The logistic regression model was performed in a step-forward fashion and clinical and molecular variables with a p-value <0.20 in the univariate analysis were selected to build a final model, which was then further adjusted for important clinical variables.

The Kaplan–Meier method was used to estimate disease-free survival (DFS) and overall survival (OS) of patients, and the log-rank test was used to examine the differences between groups. The DFS was defined as the time interval between the date of the initial treatment and the date of diagnosis of the first recurrence or tumor progression or the last date of follow-up if recurrence or progression was not observed. The OS was defined as the time interval between the date of the initial treatment and the date of death or the last date of follow-up if the patient was alive. Cox proportional hazard regression analysis was performed in a step-forward fashion to estimate the hazard ratios (HR); the multivariate Cox regression model included clinical and molecular variables, which presented a p-value <0.20 in the univariate analysis and were selected to build a final model. The final model was then further adjusted for clinically important variables. A p-value <0.05 was necessary to determine statistically significant differences. All statistical analyses were performed using SPSS statistics 23.0.

## References

[1] Ferlay J, Steliarova-Foucher E, Lortet-Tieulent J, Rosso S, Coebergh JW, Comber H, Forman D, Bray F (2013). Cancer incidence and mortality patterns in Europe: estimates for 40 countries in 2012. European journal of cancer.

[2] Jemal A, Bray F, Center MM, Ferlay J, Ward E, Forman D (2011). Global cancer statistics. CA: a cancer journal for clinicians.

[3] de Souza Viana L, de Aguiar Silva FC, Andrade Dos Anjos Jacome A, Calheiros Campelo Maia D, Duarte de Mattos M, Arthur Jacinto A, Elias Mamere A, Boldrini Junior D, de Castro Capuzzo R, Roberto Santos C, Lopes Carvalho A. (2016). Efficacy and safety of a cisplatin and paclitaxel induction regimen followed by chemoradiotherapy for patients with locally advanced head and neck squamous cell carcinoma. Head & neck.

[4] Argiris A, Karamouzis MV, Raben D, Ferris RL (2008). Head and neck cancer. Lancet.

[5] Haddad RI, Shin DM (2008). Recent advances in head and neck cancer. The New England journal of medicine.

[6] Mendenhall WM, Mendenhall CM, Malyapa RS, Palta JR, Mendenhall NP (2008). Re-irradiation of head and neck carcinoma. American journal of clinical oncology.

[7] He Q, Chen Z, Cabay RJ, Zhang L, Luan X, Chen D, Yu T, Wang A, Zhou X (2016). microRNA-21 and microRNA-375 from oral cytology as biomarkers for oral tongue cancer detection. Oral oncology.

[8] Di Leva G, Garofalo M, Croce CM (2014). MicroRNAs in cancer. Annual review of pathology.

[9] Summerer I, Hess J, Pitea A, Unger K, Hieber L, Selmansberger M, Lauber K, Zitzelsberger H (2015). Integrative analysis of the microRNA-mRNA response to radiochemotherapy in primary head and neck squamous cell carcinoma cells. BMC genomics.

[10] Chen X, Sturgis EM, Wang C, Cao X, Li Y, Wei Q, Li G (2016). Significance of microRNA-related variants in susceptibility to recurrence of oropharyngeal cancer patients after definitive radiotherapy. Oncotarget.

[11] Manikandan M, Deva Magendhra Rao AK, Arunkumar G, Manickavasagam M, Rajkumar KS, Rajaraman R, Munirajan AK (2016). Oral squamous cell carcinoma: microRNA expression profiling and integrative analyses for elucidation of tumourigenesis mechanism. Molecular cancer.

[12] Lamperska KM, Kozlowski P, Kolenda T, Teresiak A, Blizniak R, Przybyla W, Masternak MM, Golusinski P, Golusinski W (2016). Unpredictable changes of selected miRNA in expression profile of HNSCC. Cancer biomarkers.

[13] Wong N, Khwaja SS, Baker CM, Gay HA, Thorstad WL, Daly MD, Lewis JS, Wang X. (2016). Prognostic microRNA signatures derived from The Cancer Genome Atlas for head and neck squamous cell carcinomas. Cancer medicine.

[14] Vandenboom Ii TG, Li Y, Philip PA, Sarkar FH (2008). MicroRNA and Cancer: Tiny Molecules with Major Implications. Current genomics.

[15] Selcuklu SD, Donoghue MT, Spillane C (2009). miR-21 as a key regulator of oncogenic processes. Biochemical Society transactions.

[16] Cao P, Zhou L, Zhang J, Zheng F, Wang H, Ma D, Tian J (2013). Comprehensive expression profiling of microRNAs in laryngeal squamous cell carcinoma. Head & neck.

[17] Ren W, Qiang C, Gao L, Li SM, Zhang LM, Wang XL, Dong JW, Chen C, Liu CY, Zhi KQ (2014). Circulating microRNA-21 (MIR-21) and phosphatase and tensin homolog (PTEN) are promising novel biomarkers for detection of oral squamous cell carcinoma. Biomarkers.

[18] Wang Y, Zhu Y, Lv P, Li L (2015). The role of miR-21 in proliferation and invasion capacity of human tongue squamous cell carcinoma in vitro. International journal of clinical and experimental pathology.

[19] Hu A, Huang JJ, Xu WH, Jin XJ, Li JP, Tang YJ, Huang XF, Cui HJ, Sun GB (2014). miR-21 and miR-375 microRNAs as candidate diagnostic biomarkers in squamous cell carcinoma of the larynx: association with patient survival. American journal of translational research.

[20] Chang SS, Jiang WW, Smith I, Poeta LM, Begum S, Glazer C, Shan S, Westra W, Sidransky D, Califano JA (2008). MicroRNA alterations in head and neck squamous cell carcinoma. International journal of cancer.

[21] Wong TS, Liu XB, Wong BY, Ng RW, Yuen AP, Wei WI (2008). Mature miR-184 as Potential Oncogenic microRNA of Squamous Cell Carcinoma of Tongue. Clin Cancer Res.

[22] Zhang X, Gee H, Rose B, Lee CS, Clark J, Elliott M, Gamble JR, Cairns MJ, Harris A, Khoury S, Tran N (2015). Regulation of the tumour suppressor PDCD4 by miR-499 and miR-21 in oropharyngeal cancers. BMC cancer.

[23] Li J, Huang H, Sun L, Yang M, Pan C, Chen W, Wu D, Lin Z, Zeng C, Yao Y, Zhang P, Song E (2009). MiR-21 indicates poor prognosis in tongue squamous cell carcinomas as an apoptosis inhibitor. Clin Cancer Res.

[24] Chang YC, Jan CI, Peng CY, Lai YC, Hu FW, Yu CC (2015). Activation of microRNA-494-targeting Bmi1 and ADAM10 by silibinin ablates cancer stemness and predicts favourable prognostic value in head and neck squamous cell carcinomas. Oncotarget.

[25] Romano G, Acunzo M, Garofalo M, Di Leva G, Cascione L, Zanca C, Bolon B, Condorelli G, Croce CM (2012). MiR-494 is regulated by ERK1/2 and modulates TRAIL-induced apoptosis in non-small-cell lung cancer through BIM down-regulation. Proceedings of the National Academy of Sciences of the United States of America.

[26] Shen PF, Chen XQ, Liao YC, Chen N, Zhou Q, Wei Q, Li X, Wang J, Zeng H (2014). MicroRNA-494-3p targets CXCR4 to suppress the proliferation, invasion, and migration of prostate cancer. The Prostate.

[27] Kim WK, Park M, Kim YK, Tae YK, Yang HK, Lee JM, Kim H (2011). MicroRNA-494 downregulates KIT and inhibits gastrointestinal stromal tumor cell proliferation. Clin Cancer Res.

[28] Sun HB, Chen X, Ji H, Wu T, Lu HW, Zhang Y, Li H, Li YM (2014). miR494 is an independent prognostic factor and promotes cell migration and invasion in colorectal cancer by directly targeting PTEN. International journal of oncology.

[29] Lee DJ, Koch WM, Yoo G, Lango M, Reed A, Califano J, Brennan JA, Westra WH, Zahurak M, Sidransky D (1997). Impact of chromosome 14q loss on survival in primary head and neck squamous cell carcinoma. Clin Cancer Res.

[30] Liborio-Kimura TN, Jung HM, Chan EK (2015). miR-494 represses HOXA10 expression and inhibits cell proliferation in oral cancer. Oral oncology.

[31] Ries J, Vairaktaris E, Agaimy A, Kintopp R, Baran C, Neukam FW, Nkenke E (2014). miR-186, miR-3651 and miR-494: potential biomarkers for oral squamous cell carcinoma extracted from whole blood. Oncology reports.

[32] Kumar P, Anaya J, Mudunuri SB, Dutta A (2014). Meta-analysis of tRNA derived RNA fragments reveals that they are evolutionarily conserved and associate with AGO proteins to recognize specific RNA targets. BMC biology.

[33] Kumar P, Mudunuri SB, Anaya J, Dutta A (2015). tRFdb: a database for transfer RNA fragments. Nucleic acids research.

[34] Armstrong DA, Green BB, Seigne JD, Schned AR, Marsit CJ (2015). MicroRNA molecular profiling from matched tumor and bio-fluids in bladder cancer. Molecular cancer.

[35] Tang Y, Lin Y, Li C, Hu X, Liu Y, He M, Luo J, Sun G, Wang T, Li W, Guo M (2015). MicroRNA-720 promotes in vitro cell migration by targeting Rab35 expression in cervical cancer cells. Cell & bioscience.

[36] Nonaka R, Miyake Y, Hata T, Kagawa Y, Kato T, Osawa H, Nishimura J, Ikenaga M, Murata K, Uemura M, Okuzaki D, Takemasa I, Mizushima T, Yamamoto H, Doki Y, Mori M (2015). Circulating miR-103 and miR-720 as novel serum biomarkers for patients with colorectal cancer. International journal of oncology.

[37] Jones CI, Zabolotskaya MV, King AJ, Stewart HJ, Horne GA, Chevassut TJ, Newbury SF (2012). Identification of circulating microRNAs as diagnostic biomarkers for use in multiple myeloma. British journal of cancer.

[38] Lin Y, Zeng Y, Zhang F, Xue L, Huang Z, Li W, Guo M (2013). Characterization of microRNA expression profiles and the discovery of novel microRNAs involved in cancer during human embryonic development. PloS one.

[39] Li LZ, Zhang CZ, Liu LL, Yi C, Lu SX, Zhou X, Zhang ZJ, Peng YH, Yang YZ, Yun JP (2014). miR-720 inhibits tumor invasion and migration in breast cancer by targeting TWIST1. Carcinogenesis.

[40] Nordentoft I, Birkenkamp-Demtroder K, Agerbaek M, Theodorescu D, Ostenfeld MS, Hartmann A, Borre M, Orntoft TF, Dyrskjot L (2012). miRNAs associated with chemo-sensitivity in cell lines and in advanced bladder cancer. BMC medical genomics.

[41] Zhou M, Liu Z, Zhao Y, Ding Y, Liu H, Xi Y, Xiong W, Li G, Lu J, Fodstad O, Riker AI, Tan M (2010). MicroRNA-125b confers the resistance of breast cancer cells to paclitaxel through suppression of pro-apoptotic Bcl-2 antagonist killer 1 (Bak1) expression. The Journal of biological chemistry.

[42] Zhu DX, Zhu W, Fang C, Fan L, Zou ZJ, Wang YH, Liu P, Hong M, Miao KR, Liu P, Xu W, Li JY (2012). miR-181a/b significantly enhances drug sensitivity in chronic lymphocytic leukemia cells via targeting multiple anti-apoptosis genes. Carcinogenesis.

[43] Altmae S, Martinez-Conejero JA, Esteban FJ, Ruiz-Alonso M, Stavreus-Evers A, Horcajadas JA, Salumets A (2013). MicroRNAs miR-30b, miR-30d, and miR-494 regulate human endometrial receptivity. Reproductive sciences.

[44] https://www.r-project.org.

[45] http://bioconductor.org/packages/release/bioc/html/aroma.light.html.

[46] http://bioconductor.org/packages/release/bioc/html/RankProd.html.

[47] http://bioconductor.org/packages/release/bioc/html/ComplexHeatmap.html.

[48] Livak KJ, Schmittgen TD (2001). Analysis of relative gene expression data using real-time quantitative PCR and the 2(-Delta Delta C(T)) Method. Methods.

